# Artificial intelligence derived large language model in decision-making process in uveitis

**DOI:** 10.1186/s40942-024-00581-1

**Published:** 2024-09-11

**Authors:** Inès Schumacher, Virginie Manuela Marie Bühler, Damian Jaggi, Janice Roth

**Affiliations:** 1 Department of Ophthalmology, Inselspital, University Hospital of Bern, Bern, Switzerland; 2 Moorfields Eye Hospital NHS Foundation Trust, City Road, EC1V 2 London, PD UK

## Abstract

**Background:**

Uveitis is the ophthalmic subfield dealing with a broad range of intraocular inflammatory diseases. With the raising importance of LLM such as ChatGPT and their potential use in the medical field, this research explores the strengths and weaknesses of its applicability in the subfield of uveitis.

**Methods:**

A series of highly clinically relevant questions were asked three consecutive times (attempts 1, 2 and 3) of the LLM regarding current uveitis cases. The answers were classified on whether they were accurate and sufficient, partially accurate and sufficient or inaccurate and insufficient. Statistical analysis included descriptive analysis, normality distribution, non-parametric test and reliability tests. References were checked for their correctness in different medical databases.

**Results:**

The data showed non-normal distribution. Data between subgroups (attempts 1, 2 and 3) was comparable (Kruskal-Wallis H test, *p*-value = 0.7338). There was a moderate agreement between attempt 1 and attempt 2 (Cohen’s kappa, ĸ = 0.5172) as well as between attempt 2 and attempt 3 (Cohen’s kappa, ĸ = 0.4913). There was a fair agreement between attempt 1 and attempt 3 (Cohen’s kappa, ĸ = 0.3647). The average agreement was moderate (Cohen’s kappa, ĸ = 0.4577). Between the three attempts together, there was a moderate agreement (Fleiss’ kappa, ĸ = 0.4534). A total of 52 references were generated by the LLM. 22 references (42.3%) were found to be accurate and correctly cited. Another 22 references (42.3%) could not be located in any of the searched databases. The remaining 8 references (15.4%) were found to exist, but were either misinterpreted or incorrectly cited by the LLM.

**Conclusion:**

Our results demonstrate the significant potential of LLMs in uveitis. However, their implementation requires rigorous training and comprehensive testing for specific medical tasks. We also found out that the references made by ChatGPT 4.o were in most cases incorrect. LLMs are likely to become invaluable tools in shaping the future of ophthalmology, enhancing clinical decision-making and patient care.

## Background

Large language models (LLMs) such as ChatGPT (OpenAI, San Francisco, California, USA) or Claude (Anthropic San Francisco, California, USA) are computational models able to process language and generate general-purpose language through algorithms. In recent years, using LLMs has become a key feature in different areas and for different tasks. In medicine, LLMs are increasingly being studied and their effectiveness tested in various specialties. Particularly in ophthalmology, their utility has been tested in a variety of tasks including medical assistance, patient information and medical education [1–5]. Despite some approaches, the clinical applicability of LLMs in the decision-making process for patients with uveitis remains underexplored. Uveitis represents a complex ophthalmic subfield dealing with a broad range of intraocular inflammatory diseases and interferes with other medical subspecialties. Therefore, efficient and accurate LLMs could support clinicians in their clinical daily routine. However, the accuracy and veracity of LLM-generated responses are a critical issue. With increasing worldwide use and open access, the need for a systematic validation of certain aspects of these models is compulsory. It is crucial to identify potential limitations to prevent the unchecked spread of inaccurate clinical information. In this study, we aimed to evaluate the accuracy and consistency of a LLM in assisting ophthalmologists with the management of common uveitis cases and related questions.

## Methods

The authors designed a series of 10 common clinical uveitis cases, namely HLA-B27 uveitis, Birdshot chorioretinopathy, Vogt-Koyanagi-Harada disease, acute retinal necrosis, juvenile idiopathic arthritis with bilateral anterior uveitis, Fuchs’ uveitis syndrome, cytomegalovirus retinitis, Behçet disease, intermediated uveitis associated with multiple sclerosis and herpes simplex keratouveitis. The LLM under scrutiny were the paid version of ChatGPT 4.o (2024) by OpenAI. We have used the following prompt: “Please answer the following questions in a highly specialized medical language as if you were an expert in uveitis talking to another expert in uveitis, giving percentages, figures and references where you feel it is relevant.”

The LLM was asked a series of five questions, each covering key aspects of clinical management: diagnosis, further investigations, treatment, follow-up and prognosis for a specific disease. To assess consistency and variability in LLM responses, each question was presented three times in separate sessions. This approach generated three unique responses per question, resulting in a total of 15 responses for analysis per case. The specific questions were as follows.

- What is the diagnosis?
- Are further examinations needed and if yes, why and what are they?
- What is the treatment?
- When should the next appointment be scheduled?
- What is the prognosis?

Two uveitis specialists acted as independent graders, assessing the accuracy and sufficiency of ChatGPT responses. Their assessment was based on the clinical information provided and the specific questions asked. Answers were classified as ‘accurate and sufficient’ if they were both correct and comprehensive, ‘partially accurate and sufficient’ when they contained minor inaccuracies but still provided substantial and understandable information and ‘inaccurate’ when they were entirely incorrect or contained critical errors making them unreliable. The resulting evaluation grids were compared and in case of inter-grader variability, a common agreement discussed.

The references provided by the LLM were independently verified. A comprehensive search was conducted across multiple medical databases, including PubMed, MEDLINE, Cochrane Library, Embase, Scopus, Web of Science, and Google Scholar, to confirm the accuracy and existence of the cited sources.

Statistical analysis was conducted with Python 3.12.3 Descriptive analysis (including frequency, means and mode) and normality distribution test (Shapiro–Wilk) were done. A non-parametric Kruskal–Wallis H test was subsequently performed, given the abnormal distribution of the data, to compare average scores across the three attempts. Reliability test was performed by measuring Cohen’s kappa and Fleiss’ kappa coefficient. A *p*-value of less than 0.05 was considered statistically significant.

## Results

A total of 150 answers were generated (10 cases, 5 questions, each 3 times). Frequency distribution of the three attempts are outlined in Table 1. Statistical analysis of the data revealed complex patterns of agreement and variability. The Shapiro-Wilk test indicated a non-normal distribution and therefore non-parametric methods were used for further analysis. The Kruskal-Wallis H test yielded a *p*-value of 0.7338, which was not statistically significant, suggesting no substantial differences across the three attempts for each question.

**Table 1:** The common agreement of evaluation of answers generated by ChatGPT are summarized. Every question was asked 3 consecutive times (= “attempts 1, 2 and 3”)

Cases and Questions	ChatGPT attempt 1	ChatGPT attempt 2	ChatGPT attempt 3
1. HLA-B27 Uveitis: What is the diagnosis?	1	1	1
1. HLA-B27 Uveitis: Are further examinations needed and if yes, why and what are they?	1	1	1
1. HLA-B27 Uveitis: What is the treatment?	1	1	1
1. HLA-B27 Uveitis: When should the next appointment be scheduled?	1	1	1
1. HLA-B27 Uveitis: What is the prognosis?	1	1	1
2. Birdshort Chorioretinopathy: What is the diagnosis?	1	1	1
2. Birdshort Chorioretinopathy: Are further examinations needed and if yes, why and what are they?	1	1	1
2. Birdshort Chorioretinopathy: What is the treatment?	1	1	2
2. Birdshort Chorioretinopathy: When should the next appointment be scheduled?	1	1	1
2. Birdshort Chorioretinopathy: What is the prognosis?	2	2	2
3. Vogt-Koyanagi-Harada disease: What is the diagnosis?	1	1	1
3. Vogt-Koyanagi-Harada disease: Are further examinations needed and if yes, why and what are they?	1	1	1
3. Vogt-Koyanagi-Harada disease: What is the treatment?	1	1	1
3. Vogt-Koyanagi-Harada disease: When should the next appointment be scheduled?	1	1	1
3. Vogt-Koyanagi-Harada disease: What is the prognosis?	1	1	1
4. Acute Retinal Necrosis (ARN): What is the diagnosis?	1	1	1
4. Acute Retinal Necrosis (ARN): Are further examinations needed and if yes, why and what are they?	1	1	1
4. Acute Retinal Necrosis (ARN): What is the treatment?	1	1	2
4. Acute Retinal Necrosis (ARN): When should the next appointment be scheduled?	1	1	1
4. Acute Retinal Necrosis (ARN): What is the prognosis?	1	2	1
5. Juvenile Idiopathic Arthritis (JIA) with bilateral Uveitis: What is the diagnosis?	1	1	1
5. Juvenile Idiopathic Arthritis (JIA) with bilateral Uveitis: Are further examinations needed and if yes, why and what are they?	1	2	2
5. Juvenile Idiopathic Arthritis (JIA) with bilateral Uveitis: What is the treatment?	2	1	1
5. Juvenile Idiopathic Arthritis (JIA) with bilateral Uveitis: When should the next appointment be scheduled?	2	2	2
5. Juvenile Idiopathic Arthritis (JIA) with bilateral Uveitis: What is the prognosis?	2	2	2
6. Fuchs’ heterochromic Uveitis: What is the diagnosis?	1	1	1
6. Fuchs’ heterochromic Uveitis: Are further examinations needed and if yes, why and what are they?	3	3	2
6. Fuchs’ heterochromic Uveitis: What is the treatment?	1	1	1
6. Fuchs’ heterochromic Uveitis: When should the next appointment be scheduled?	1	1	1
6. Fuchs’ heterochromic Uveitis: What is the prognosis?	1	1	1
7. Cytomegalovirus (CMV) Retinitis: What is the diagnosis?	1	1	1
7. Cytomegalovirus (CMV) Retinitis: Are further examinations needed and if yes, why and what are they?	1	1	3
7. Cytomegalovirus (CMV) Retinitis: What is the treatment?	1	1	1
7. Cytomegalovirus (CMV) Retinitis: When should the next appointment be scheduled?	1	1	1
7. Cytomegalovirus (CMV) Retinitis: What is the prognosis?	1	1	1
8. Behçet disease: What is the diagnosis?	1	1	1
8. Behçet disease: Are further examinations needed and if yes, why and what are they?	2	1	1
8. Behçet disease: What is the treatment?	1	1	1
8. Behçet disease: When should the next appointment be scheduled?	2	1	1
8. Behçet disease: What is the prognosis?	1	1	1
9. Multiple sclerosis: What is the diagnosis?	1	1	1
9. Multiple sclerosis: Are further examinations needed and if yes, why and what are they?	2	2	1
9. Multiple sclerosis: What is the treatment?	2	1	1
9. Multiple sclerosis: When should the next appointment be scheduled?	1	1	1
9. Multiple sclerosis: What is the prognosis?	1	1	1
10. HSV1 keratouveitis: What is the diagnosis?	1	1	1
10. HSV1 keratouveitis: Are further examinations needed and if yes, why and what are they?	1	1	1
10. HSV1 keratouveitis: What is the treatment?	2	1	2
10. HSV1 keratouveitis: When should the next appointment be scheduled?	1	1	1
10. HSV1 keratouveitis: What is the prognosis?	1	1	1

**Legend: 1 = accurate and sufficient; 2 = partially accurate and sufficient; 3 = inaccurate and insufficient**

To assess the agreement between pairs of attempts, Cohen’s kappa coefficients were calculated. Moderate agreement was observed between attempts 1 and 2 (κ = 0.5172), as well as between attempts 2 and 3 (κ = 0.4913). However, the agreement between attempts 1 and 3 was only fair (κ = 0.3647). The average Cohen’s kappa score (κ = 0.4577) indicated an overall moderate level of agreement. To evaluate the consistency across all three attempts simultaneously, Fleiss’ kappa was employed, resulting in a score of κ = 0.4534, which also signifies moderate agreement.

The frequency distribution of subgroups are outlined in Tables 2 and 3. The answers about diagnosis were the most accurate, followed by follow-up, treatment, prognosis and further investigation in descending order. ChatGPT 4.o (2024) was able to give accurate and sufficient answers in > 80% of cases. A range of 12–18% of answers were accurate but insufficient. In 2% of cases, the answers were inaccurate and insufficient. The answers concerning HLA-B27 and VKH syndrome were the most accurate and the least accurate for JIA (Table 4).

**Table 2:** Frequency of distribution for each ChatGPT attempt. In attempt 1, 80% (*n* = 40) of answers were accurate and sufficient, 18% (*n* = 9) accurate but insufficient and 2% were inaccurate and insufficient. In attempt 2, 86% (*n* = 43) of answers were accurate and sufficient, 12% (*n* = 6) were accurate but insufficient and 2% (*n* = 1) were inaccurate and insufficient. In attempt 3, 82% (*n* = 41) of answers were accurate and sufficient, 16% (*n* = 8) were accurate but insufficient and 2% (*n* = 1) were inaccurate and insufficient

Accuracy	ChatGPT (attempt 1)	ChatGPT (attempt 2)	ChatGPT (attempt 3)
1	40	43	41
2	9	6	8
3	1	1	1

**Table 3:** Frequency of distribution for each question. The accuracy of answers was best for the diagnosis, followed by follow-up, treatment, prognosis and further investigation in descending order

Accuracy	Diagnosis	Investigations	Treatment	Follow-Up	Prognosis
1	30	19	24	26	23
2	0	8	6	4	7
3	0	3	0	0	0

**Table 4:** Frequency of distribution for each case. The accuracy was best for HLA-B27 and VKH syndrome and worse for Fuchs’, JIA and CMV

Accuracy	HLA-B27	Birdshot	VKH	ARN	JIA
1	15	11	15	13	6
2	0	4	0	2	9
3	0	0	0	0	0
	**Fuchs’**	**CMV**	**Behçet**	**MS**	**HSV1**
1	12	14	13	12	13
2	1	0	2	3	2
3	2	1	0	0	0

A total of 52 references were generated by the LLM. Upon rigorous verification, 22 references (42.3%) were found to be accurate and correctly cited. Another 22 references (42.3%) could not be located in any of the searched databases, suggesting they may be a hallucination. The remaining 8 references (15.4%) were found to exist, but were either misinterpreted or incorrectly cited by the LLM. To give an example, the Jabs et al.’s Standardization of Uveitis Nomenclature (SUN) Working Group was correctly cited at three different occasions (attempt 1 and 2 of the case of intermediate uveitis associated with multiple sclerosis and in an attempt of the case of Fuchs’ uveitis syndrome) [6]. Yet, the paper about “Cytomegalovirus retinitis in patients with acquired immunodeficiency syndrome” that was pretended published in the journal “Ophthalmology” in 2010 with the reference 117(6):1232–1239 of the same author could not be tracked. Interestingly, a paper named “Cytomegalovirus retinitis and acquired immunodeficiency syndrome” from the same author was found in the journal “Archives of Ophthalmology” and published in 1989 [7].

## Discussion

This study aimed to evaluate the accuracy and consistency of a LLM in assisting ophthalmologists with the management of common uveitis cases. These findings collectively suggest that while the LLM demonstrated some consistency in its responses across multiple attempts, there was still notable variability. This emphasizes the importance of considering multiple outputs when relying on such systems for clinical information and highlights the need for human expertise in interpreting and validating AI-generated medical advice. The overall moderate level of agreement observed indicates that while the LLM shows promise in providing consistent information, there is still room for improvement in its reliability and reproducibility in clinical contexts. The difference in accuracy between the single attempts could suggest or even underline that the answers generated are not based on comprehension and knowledge but precisely through automated algorithms that fill out “blanks” randomly. The resulting poor reliability and reproducibility were observed before and seem to be a major problem of LLMs in that context [3]. One way to further fine-tune the use of LLMs and is of emerging importance is the use of prompts or commands in order to optimize the potential and benefits of AI systems [8]. Prompt engineering is the strategic and research-driven usage of LLMs and can maximize its accuracy, relevance and utility on one hand but also confuse models on the other hand, if it is not applied correctly [8].

Within subgroups of questions, ChatGPT shows variability of accuracy. We found excellent potential in correctly identifying diagnosis with 100% in our series. This agrees with a diagnostic success rate of 66% and up found in other studies [4, 9]. ChatGPT correctly diagnosed > 90% of case reports in another work [10]. In a systematic review of Jacquot et al., a LLM classification accuracy of 93–99% and a sensitivity of at least 80% for identifying most probable etiologies for uveitis were found [5]. We have also found ChatGPT answers to be less accurate and complete in determining and explaining the need and content for further investigations. The fact that ChatGPT answers can be incomplete is nothing new and seems to depend on the complexity of a given task^8 10^. This is also our hypothesis, meaning that answers requiring a certain extent of information in order to be answered correctly will logically be more prone to errors.

The variability in accuracy was also observed within subgroups of cases. Responses were fully correct for HLA-B27-associated uveitis management but showed significant variability for JIA-related bilateral anterior uveitis. The reason for this inconsistency was unclear. We examined ChatGPT’s citations to investigate potential correlations. Initially, there seemed to be a connection. For example, HLA-B27-associated uveitis management had up to 4 references, while JIA-related uveitis had none. However, analysis of other sections revealed that the presence or absence of references alone couldn’t explain answer variability. In the VKH case’s first attempt, answers were sufficient and accurate without references. Conversely, in the CMV retinitis case’s third attempt, the answer about further examinations was inadequate, despite citing the same reference as the first attempt’s adequate response.

Possible reasons for this variability could include inconsistencies in training data, sensitivity to prompt phrasing, the stochastic nature of language models (randomness in their outputs, which could contribute to variability across attempts), or limitations in maintaining context across queries. However, future research is needed to establish the precise causes of these inconsistencies and to develop more reliable AI-based medical information systems.

Our analysis revealed significant concerns regarding the reliability of the LLM’s citation practices, with less than half of the provided references being both accurate and verifiable. The high proportion of unfound and misrepresented sources underscores the importance of human oversight in verifying AI-generated academic content [12, 13]. This making-up of references has been already described with the previous versions of ChatGPT as well as in other LLMs and is well known under the term of AI-hallucinations, which are defined as ‘inventions’ of some information by the chatbot [11, 12]. Even though we could not find any study describing the rate of AI-hallucinations with ChatGPT 4.o, it seems reasonable to think that they still exist and cause a serious challenge for the generalised use of AI in the daily medical practice.

The implementation of LLMs in clinical practice requires careful consideration of their limitations and variability. While they show promise in providing consistent and accurate information in many cases, the presence of inaccuracies and inconsistencies necessitates ongoing human oversight and validation. Future research should focus on improving the reliability and reproducibility of LLM outputs in clinical contexts, as well as developing robust methods for integrating these tools into medical practice safely and effectively.

## Conclusion

Our results demonstrate the potential of LLMs in ophthalmology. However, careful implementation is essential, requiring extensive training and testing for specific medical tasks. As these technologies continue to develop, it is likely that LLMs will become a valuable tool in shaping the future of ophthalmology, supporting both clinical decision making and patient care. Future, large-scale and real-life research should focus on ways to improve accuracy and repeatability of LLM outputs such as with the use of prompt engineering.

## Data Availability

No datasets were generated or analysed during the current study.
